# Supplementary material for: In the Search for Novel, Isoflavone-Rich Functional Foods—Comparative Studies of Four Clover Species Sprouts and Their Chemopreventive Potential for Breast and Prostate Cancer
Source: Pharmaceuticals (Basel). 2022 Jun 28;15(7):806. doi: 10.3390/ph15070806 (PMC9319781; doi:10.3390/ph15070806)
Supplement: Supplementary file 1 [file pharmaceuticals-15-00806-s001.zip › pharmaceuticals-1789287-supplementary.pdf]

Table S1. Statistical differences for quantitative analysis of isoflavones in four clover species sprouts

GENISTEIN	RCS	RC3	RC5	RC7	RC10	WCS	WC3	WC5	WC7	WC10	PCS	PC3	PC5	PC7	PC10	CCS	CC3	CC5	CC7	CC10
RCS																				
RC3																				
RC5																				
RC7																				
RC10																				
WCS																				
WC3																				
WC5																				
WC7																				
WC10																				
PCS																				
PC3																				
PC5																				
PC7																				
PC10																				
CCS																				
CC3																				
CC5																				
CC7																				
CC10																				
		Not significant			P<0.05			P<0.01			P<0.001									
DAIDZEIN	RCS	RC3	RC5	RC7	RC10	WCS	WC3	WC5	WC7	WC10	PCS	PC3	PC5	PC7	PC10	CCS	CC3	CC5	CC7	CC10
RCS																				
RC3																				
RC5																				
RC7																				
RC10																				
WCS																				
WC3																				
WC5																				
WC7																				
WC10																				
PCS																				
PC3																				
PC5																				
PC7																				
PC10																				
CCS																				
CC3																				
CC5																				
CC7																				
CC10																				
		Not significant			P<0.05			P<0.01			P<0.001									
ONONIN	RCS	RC3	RC5	RC7	RC10	WCS	WC3	WC5	WC7	WC10	PCS	PC3	PC5	PC7	PC10	CCS	CC3	CC5	CC7	CC10
RCS																				
RC3																				
RC5																				
RC7																				
RC10																				

WCS																				
WC3																				
WC5																				
WC7																				
WC10																				
PCS																				
PC3																				
PC5																				
PC7																				
PC10																				
CCS																				
CC3																				
CC5																				
CC7																				
CC10																				
		Not significant			P<0.05			P<0.01			P<0.001									

FORMONONETIN	RCS	RC3	RC5	RC7	RC10	WCS	WC3	WC5	WC7	WC10	PCS	PC3	PC5	PC7	PC10	CCS	CC3	CC5	CC7	CC10
RCS																				
RC3																				
RC5																				
RC7																				
RC10																				
WCS																				
WC3																				
WC5																				
WC7																				
WC10																				
PCS																				
PC3																				
PC5																				
PC7																				
PC10																				
CCS																				
CC3																				
CC5																				
CC7																				
CC10																				
		Not significant			P<0.05			P<0.01			P<0.001									

DAIDZIN	RCS	RC3	RC5	RC7	RC10	WCS	WC3	WC5	WC7	WC10	PCS	PC3	PC5	PC7	PC10	CCS	CC3	CC5	CC7	CC10
RCS																				
RC3																				
RC5																				
RC7																				
RC10																				
WCS																				
WC3																				
WC5																				
WC7																				
WC10																				
PCS																				
PC3																				
PC5																				
PC7																				
PC10																				

CCS																				
CC3																				
CC5																				
CC7																				
CC10																				
		Not significant			P<0.05			P<0.01			P<0.001									

Table S2. Statistical differences for cytotoxic activity of four clover species sprouts (500 µg/mL) to breast cancer and normal cells

MCF10A	RCS	RC3	RC5	RC7	RC10	WCS	WC3	WC5	WC7	WC10	PCS	PC3	PC5	PC7	PC10	CCS	CC3	CC5	CC7	CC10
RCS																				
RC3																				
RC5																				
RC7																				
RC10																				
WCS																				
WC3																				
WC5																				
WC7																				
WC10																				
PCS																				
PC3																				
PC5																				
PC7																				
PC10																				
CCS																				
CC3																				
CC5																				
CC7																				
CC10																				
		Not significant			P<0.05			P<0.01			P<0.001									
MDA-MB-231	RCS	RC3	RC5	RC7	RC10	WCS	WC3	WC5	WC7	WC10	PCS	PC3	PC5	PC7	PC10	CCS	CC3	CC5	CC7	CC10
RCS																				
RC3																				
RC5																				
RC7																				
RC10																				
WCS																				
WC3																				
WC5																				
WC7																				
WC10																				
PCS																				
PC3																				
PC5																				
PC7																				
PC10																				
CCS																				
CC3																				
CC5																				
CC7																				
CC10																				
		Not significant			P<0.05			P<0.01			P<0.001									
MCF	RCS	RC3	RC5	RC7	RC10	WCS	WC3	WC5	WC7	WC10	PCS	PC3	PC5	PC7	PC10	CCS	CC3	CC5	CC7	CC10

RCS																				
RC3																				
RC5																				
RC7																				
RC10																				
WCS																				
WC3																				
WC5																				
WC7																				
WC10																				
PCS																				
PC3																				
PC5																				
PC7																				
PC10																				
CCS																				
CC3																				
CC5																				
CC7																				
CC10																				
		Not significant			P<0.05			P<0.01			P<0.001									

Table S3 Statistical differences for cytotoxic activity of four clover species sprouts (500 µg/mL) to prostate cancer and normal cells

DU145	RCS	RC3	RC5	RC7	RC10	WCS	WC3	WC5	WC7	WC10	PCS	PC3	PC5	PC7	PC10	CCS	CC3	CC5	CC7	CC10
RCS																				
RC3																				
RC5																				
RC7																				
RC10																				
WCS																				
WC3																				
WC5																				
WC7																				
WC10																				
PCS																				
PC3																				
PC5																				
PC7																				
PC10																				
CCS																				
CC3																				
CC5																				
CC7																				
CC10																				
		Not significant			P<0.05			P<0.01			P<0.001									

PC3	RCS	RC3	RC5	RC7	RC10	WCS	WC3	WC5	WC7	WC10	PCS	PC3	PC5	PC7	PC10	CCS	CC3	CC5	CC7	CC10
RCS																				
RC3																				
RC5																				
RC7																				
RC10																				
WCS																				
WC3																				
WC5																				

WC7																				
WC10																				
PCS																				
PC3																				
PC5																				
PC7																				
PC10																				
CCS																				
CC3																				
CC5																				
CC7																				
CC10																				
		Not significant			P<0.05			P<0.01			P<0.001									

LNCaP	RCS	RC3	RC5	RC7	RC10	WCS	WC3	WC5	WC7	WC10	PCS	PC3	PC5	PC7	PC10	CCS	CC3	CC5	CC7	CC10
RCS																				
RC3																				
RC5																				
RC7																				
RC10																				
WCS																				
WC3																				
WC5																				
WC7																				
WC10																				
PCS																				
PC3																				
PC5																				
PC7																				
PC10																				
CCS																				
CC3																				
CC5																				
CC7																				
CC10																				
		Not significant			P<0.05			P<0.01			P<0.001									

PNT2	RCS	RC3	RC5	RC7	RC10	WCS	WC3	WC5	WC7	WC10	PCS	PC3	PC5	PC7	PC10	CCS	CC3	CC5	CC7	CC10
RCS																				
RC3																				
RC5																				
RC7																				
RC10																				
WCS																				
WC3																				
WC5																				
WC7																				
WC10																				
PCS																				
PC3																				
PC5																				
PC7																				
PC10																				
CCS																				
CC3																				
CC5																				
CC7																				
CC10																				
		Not significant			P<0.05			P<0.01			P<0.001									

Table S4 Statistical differences for antioxidant activity of four clover species sprouts

DPPH	RCS	RC3	RC5	RC7	RC10	WCS	WC3	WC5	WC7	WC10	PCS	PC3	PC5	PC7	PC10	CCS	CC3	CC5	CC7	CC10
RCS																				
RC3																				
RC5																				
RC7																				
RC10																				
WCS																				
WC3																				
WC5																				
WC7																				
WC10																				
PCS																				
PC3																				
PC5																				
PC7																				
PC10																				
CCS																				
CC3																				
CC5																				
CC7																				
CC10																				
		Not significant			P<0.05			P<0.01			P<0.001									

FRAP	RCS	RC3	RC5	RC7	RC10	WCS	WC3	WC5	WC7	WC10	PCS	PC3	PC5	PC7	PC10	CCS	CC3	CC5	CC7	CC10
RCS																				
RC3																				
RC5																				
RC7																				
RC10																				
WCS																				
WC3																				
WC5																				
WC7																				
WC10																				
PCS																				
PC3																				
PC5																				
PC7																				
PC10																				
CCS																				
CC3																				
CC5																				
CC7																				
CC10																				
		Not significant			P<0.05			P<0.01			P<0.001									

Table S5. Correlation weights for the pairs of parameters based on PCA model.

Pairs of correlated parameters		Correlation weights
PNT2	MCF7	0.223
MCF10A	MCF7	0.222
PNT2	MCF10A	0.220
MCF10A	MDA-MB-231	0.202
MDA-MB-231	MCF7	0.202
PNT2	MDA-MB-231	0.202
Ononin	Daidzin	0.188
Genistein	FRAP	0.176
Formononetin	Daidzin	0.171
Ononin	Formononetin	0.167
Genistein	Daidzin	0.166
Genistein	Ononin	0.156
Daidzin	FRAP	0.154
Ononin	FRAP	0.144
Genistein	Formononetin	0.143
Formononetin	FRAP	0.132
